# Occurrence of *Babesia *spp., *Rickettsia *spp. and *Bartonella *spp. in *Ixodes ricinus *in Bavarian public parks, Germany

**DOI:** 10.1186/1756-3305-4-135

**Published:** 2011-07-15

**Authors:** Sabine Schorn, Kurt Pfister, Holger Reulen, Monia Mahling, Cornelia Silaghi

**Affiliations:** 1 Comparative Tropical Medicine and Parasitology, Ludwig-Maximilians-University, Munich, Germany; 2 Statistical Consulting Unit, Department of Statistics, Ludwig-Maximilians-University, Munich, Germany

## Abstract

**Background:**

Only limited information is available about the occurrence of ticks and tick-borne pathogens in public parks, which are areas strongly influenced by human beings. For this reason, *Ixodes ricinus *were collected in public parks of different Bavarian cities in a 2-year survey (2009 and 2010) and screened for DNA of *Babesia *spp., *Rickettsia *spp. and *Bartonella *spp. by PCR. Species identification was performed by sequence analysis and alignment with existing sequences in GenBank. Additionally, coinfections with *Anaplasma phagocytophilum *were investigated.

**Results:**

The following prevalences were detected: *Babesia *spp.: 0.4% (n = 17, including one pool of two larvae) in 2009 and 0.5 to 0.7% (n = 11, including one pool of five larvae) in 2010; *Rickettsia *spp.: 6.4 to 7.7% (n = 285, including 16 pools of 76 larvae) in 2009. DNA of *Bartonella *spp. in *I. ricinus *in Bavarian public parks could not be identified. Sequence analysis revealed the following species: *Babesia *sp. EU1 (n = 25), *B. divergens *(n = 1), *B. divergens/capreoli *(n = 1), *B. gibsoni*-like (n = 1), *R. helvetica *(n = 272), *R. monacensis *IrR/Munich (n = 12) and unspecified *R. monacensis *(n = 1). The majority of coinfections were *R. helvetica *with *A. phagocytophilum *(n = 27), but coinfections between *Babesia *spp. and *A. phagocytophilum*, or *Babesia *spp. and *R. helvetica *were also detected.

**Conclusions:**

*I. ricinus *ticks in urban areas of Germany harbor several tick-borne pathogens and coinfections were also observed. Public parks are of particularly great interest regarding the epidemiology of tick-borne pathogens, because of differences in both the prevalence of pathogens in ticks as well as a varying species arrangement when compared to woodland areas. The record of DNA of a *Babesia gibsoni*-like pathogen detected in *I. ricinus *suggests that *I. ricinus *may harbor and transmit more *Babesia *spp. than previously known. Because of their high recreational value for human beings, urban green areas are likely to remain in the research focus on public health issues.

## Background

*Ixodes *(*I*.) *ricinus*, the most common tick species in Europe, serves as an important vector for several microbial pathogens. Beside *Borrelia burgdorferi sensu lato *and the tick-borne encephalitis virus, other pathogens such as *Babesia *(*B*.) spp., Spotted Fever Group (SFG) Rickettsiae, *Anaplasma *(*A*.) *phagocytophilum*, and *Bartonella *spp. are of increasing public health interest [1].

*Babesia *spp., protozoans of the phylum Apicomplexa, have been well known pathogens in veterinary medicine since the 19^th ^century and cause babesiosis in domestic animals [2]. Human babesiosis, primarily caused by *B. microti *in the USA and by *B. divergens *in Europe, was first documented in 1957 in former Yugoslavia. Since then, several hundred clinical cases in the USA and about 40 clinical cases in Europe have been recorded. However, in Europe it occurs almost exclusively in splenectomized or otherwise immunosuppressed patients [3,4]. Symptoms are flu-like (high fever, malaise, chills, myalgia, anemia, fatigue, nausea, vomiting and diarrhoea) and the course of disease can range from mild to fatal [5]. Beside *B. microti *and *B. divergens*, clinical cases of human babesiosis caused by *B. duncani *n. sp. and a *B. divergens*-like pathogen in the USA, and by *Babesia *sp. EU1 (*B. venatorum*) in Europe have been noticed in the last 15 years [6-9]. Infections due to *B. microti *seem less acute than with *B. divergens *and infections with *Babesia *sp. EU1 are generally milder [5,10]. In previous investigations of *I. ricinus *in several European countries the species *B. divergens*, *B. microti *and *Babesia *sp. EU1 have been detected [11-18]. Prevalences in ticks range from 0.6 to 51.04% [19,20].

Pathogens of the class *α-*Proteobacteria, like *A. phagocytophilum *and certain SFG Rickettsiae, represent a potential risk for human health. *A. phagocytophilum *is the etiological agent of human granulocytic anaplasmosis (HGA), a febrile illness with malaise, myalgia and headache [21]. In the USA, HGA has an increasing incidence since the first documented clinical case in 1994, with about 1,000 cases reported to the CDC in 2008 http://www.cdc.gov/anaplasmosis[22]. In comparison, about 70 clinical cases have been documented in Europe until now [23]. *A. phagocytophilum *prevalences of 0.25 to 24.4% are found in *I. ricinus *in various European countries [24,25]. Furthermore, nine different *Rickettsia *(*R*.) spp., belonging to the SFG and able to cause rickettsiosis in humans, are detectable in Europe [26]. Of these, three pathogens (*R. helvetica, R. monacensis *and *R. massiliae*) have been detected in *I. ricinus *with prevalences from 1.7 to 31.3% [27-32]. Symptoms of SFG rickettsiosis generally include fever, headache, muscle pain, rash, local lymphadenopathy and inoculation eschar [33]. In addition, *R. helvetica *has been detected in two patients with chronic perimyocarditis in sudden cardiac death as well as attributed to one case of meningitis and eight cases with mild symptoms (fever, headache and myalgia) [34-37].

The transmission of *Bartonella *spp. by ticks, including *Bartonella henselae *as the etiological agent of cat scratch diseases, is discussed, but has so far not been fully proven [38-40]. DNA of *Bartonella henselae*, *Bartonella schoenbuchensis*-like, *Bartonella capreoli *and unspecified *Bartonella *spp. has been detected in *I. ricinus*. Prevalences varied in a wide range from 0.2 to 60% in ticks, collected from humans, dogs, deer and directly from vegetation. However, reports in Europe are very rare and data from Germany are missing to date [16,41-47].

Urban areas, especially public parks, seem to be different from woodland areas regarding the occurrence of tick-borne pathogens [48,49]. However, in European urban areas epidemiological studies on simultaneous detection of various tick-borne pathogens are rare [16,50,51]. Therefore, the aim of the study was to investigate *I. ricinus *for the occurrence of *Babesia *spp., *Rickettsia *spp. and *Bartonella *spp., and coinfections with *A. phagocytophilum*, with a special focus on urban sites. Selected Bavarian public parks, highly frequented by daily visitors and used for recreational activities, were investigated in this 2-year survey as a contribution to the current state of knowledge on the epidemiology of tick-borne pathogens in Germany.

## Methods

### Sampling sites and tick collection

**Figure 1:**
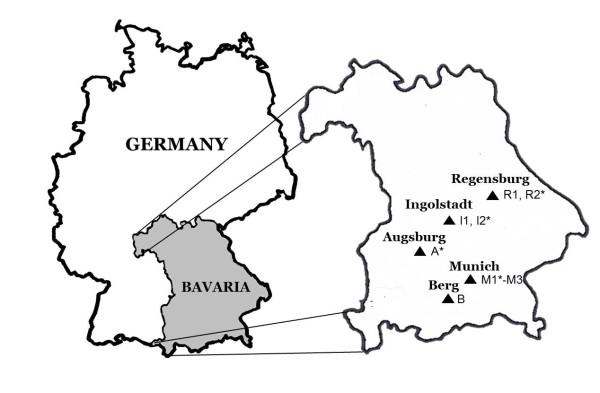
**Sampling sites**. Sites marked with * were only investigated 2009.

In 2009, nine different public parks in five Bavarian cities (Munich, Regensburg, Ingolstadt, Augsburg and Berg at the Lake Starnberg) were investigated (Figure 1). Additionally five selected sites were investigated for a second year in 2010. Seven sampling sites (M2, M3, R1, R2, I1, I2 and A) were intra-urban city parks and two sites (M1 and B) were heavily frequented suburban recreational areas in the respective cities. The vegetation of all sampling sites consists predominantly of deciduous trees, bushes and groomed lawns (with the exception of sampling area B). By comparison, two sampling sites (M3 and B) were left in a more natural state with a higher density of trees and thick and leafy undergrowth. Questing ticks were collected monthly from the vegetation (April to September 2009 and May to September 2010) by flagging and stored in 70% ethanol.

### DNA extraction

In the laboratory, ticks were identified to species level [52], after they were washed twice with distilled water and dried on a bleached pulp. Adult ticks and nymphs were handled individually and larvae in pools of up to five specimens. After the ticks were homogenized in 80 μl PBS-buffer with a 5-mm steel bead in the TissueLyser (Qiagen Hilden, Germany), DNA was extracted according to the manufacturer's tissue protocol (QIAamp DNA Mini Kit, Qiagen) in two elution steps of à 100 μl for the best quantitative DNA result. Quantitative and qualitative evaluation of DNA extraction was carried out spectrophotometrically with the NanoDrop^® ^ND-1000 system (PeqLab Erlangen, Germany).

### Molecular detection of the pathogens

In 2009, ticks from all nine sampling sites were screened for DNA of *Babesia *spp., *Rickettsia *spp., *A. phagocytophilum *and *Bartonella *spp., whereas samples from the five sites in 2010 were screened for DNA of *Babesia *spp. and *A. phagocytophilum*. Conventional PCRs with previously published primers were used for the detection of DNA of *Babesia *spp., *Rickettsia *spp. and *Bartonella *spp. (Table 1), followed by gel electrophoresis (2% Agarosegel, dyed with GelRed™ (Biotium)) [12,53-55]. The HotStarTaqPlus DNA Polymerase Kit (Qiagen, Germany) was used for molecular detection of *Babesia *spp. and *Bartonella *spp. as well as the Expand High Fidelity plus PCR System (Roche, Germany) for *Rickettsia *spp., each according to the manufacturer's protocol with a reaction volume of 50 μl, including 5.0 μl template DNA. PCRs were performed in the Thermocycler Mastercycler^® ^gradient (Eppendorf, Germany) and in every run a positive control [DNA of (i) *B. divergens *(cattle) and *B. microti *(BALB/c mice); (ii) *R. helvetica *(ticks); (iii) *Bartonella henselae *(fleas)] and PCR-clean water as a negative control were included. Detection of DNA of *A. phagocytophilum *was performed with a SYBR^®^-Green real-time PCR with primers published by Courtney et al. [56]. Detailed description of the method will be published in a separate manuscript [Schorn et al. unpublished]. For *A. phagocytophilum*, larvae were not included in the study.

**Table 1:** PCR conditions and primers used for the detection of *Babesia *spp., *Rickettsia *spp. and *Bartonella *spp.

pathogen	primer	gene (bp)	**ref**.	final primer **conc**.	Ds	As	Es	cycles
*Babesia *spp.	BJ1 5'-GTCTTGTAATTGGAATGATGG-3' BN2 5'-TAGTTTATGGTTAGGACTACG-3'	*18S rRNA* (411-452)	[12]	1 μM	94°C 30 s	55°C 30 s	72°C 40 s	40
*Rickettsia *spp.	RpCS.877p 5'-GGGGGCCTGCTCACGGCGG-3' RpCS.1258n 5'-ATTGCAAAAAGTACAGTGAACA-3'	*gltA *(380)	[54]	0,5 μM	95°C 20 s	48°C 30 s	60°C 2 min	35
	120-2788 5'-AAACAATAATCAAGGTACTGT-3' 120-3599 5'-TACTTCCGGTTACAGCAAAGT-3'	*ompB *(765)	[55]	0,5 μM	95°C 30 s	50°C 30 s	68°C 90 s	40
*Bartonella *spp.	BA325s 5'-CTTCAGATGATGATCCCAAGCCTTCTGGCG-3' BA1100as 5'-GAACCGACGACCCCCTGCTTGCAAAGCA-'3	*16S-23S rRNA *(ITS) (420-780)	[53]	1 μM	94°C 30 s	66°C 30 s	72°C 50 s	40

Ds: denaturation step; As: annealing step; Es: elongation step

Positive PCR products were purified using the QIAquick PCR Purification Kit (Qiagen) according to the manufacturer's protocol and sent for sequencing to Eurofins MWG Operon (Ebersberg, Germany). Sequencing was performed with one primer for *Rickettsia *spp. and *Bartonella *spp. and with both primers for *Babesia *spp. Results were analysed by ChromasLite^® ^and for *Babesia *spp. alignment was performed with ClustalW2 http://www.ebi.ac.uk/Tools/msa/clustalw2/. Database searches and sequence comparisons were performed with BLAST tool provided by the National Center for Biotechnology http://blast.ncbi.nlm.nih.gov/Blast.cgi.

Samples regarded as *R. monacensis *and unspecified *Rickettsia *spp. in the investigated part of the *gltA*-gene were further analysed with a PCR, targeting the *ompB*-gene for more detailed specification (Table 1).

### Statistical analysis

Logistic regression was used for statistical analysis to estimate the effect of site, month, development stage and year on the probability of infection. Logistic regression was performed accepting the minimum prevalence (in a positive pool of larvae, one specimen is assumed as positive). The variables 'April', 'September' and sampling site 'A' were removed for the statistical analysis of *Babesia *spp., because no infections of ticks were seen in these months or this sampling site and therefore the estimation of the parameters was not possible. Differences between gender, month and site were computed with a simultaneous test for general linear hypotheses based on multiple comparison of means with Tukey Contrasts [57]. p < 0.05 was regarded as significant. Statistical analysis was performed with R version 2.12.1 (R Development Core Team, 2010).

## Results

### Collection of ticks

A total of 9,107 and 4,296 *I. ricinus *were collected in 2009 and 2010, respectively.

### Molecular detection of *Babesia *spp

A total of 6,593 (2009: n = 4,459 and 2010: n = 2,134) ticks were screened for the presence of DNA of *Babesia *spp. The overall prevalences were 0.4% (n = 17, including 1 pool of 2 larvae) for 2009 and 0.5 to 0.7% (n = 11, including 1 pool of 5 larvae) for 2010 (Table 2). With regard to differences in infection there was no statistical significance of developmental stages, investigated months or sampling sites.

**Table 2:** Prevalence of tick-borne pathogens detected in *Ixodes ricinus *in Bavarian public parks, Germany, 2009/2010

					positive ticks (in %)					
					
sites	year	No. of investigated ticks			***Babesia *spp**.			***Rickettsia *spp**.		
		**A**	**N**	**L pool [n]**	**A**	**N**	**L pool [n]**	**A**	**N**	**L pool [n]**

*M1	2009	302	87	4 [14]	0	0	0	40 **(13.2)**	9 **(10.3)**	0
**M2**	2009	344	156	24 [120]	1 **(0.3)**	0	0	16 **(4.7)**	11 **(7.1)**	2 [10] **(1.7-8.3)**
	2010	267	150	3 [9]	0	0	0	n.d.	n.d.	n.d.
**M3**	2009	300	150	21 [102]	4 **(1.3)**	1 **(0.7)**	0	29 **(9.7)**	7 **(4.7)**	2 [10] **(2.0-9.8)**
	2010	272	150	23 [106]	5 **(1.8)**	0	1 [5] **(0.9-4.7)**	n.d.	n.d.	n.d.
**R1**	2009	263	129	20 [94]	0	0	0	13 **(4.9)**	9 **(7.0)**	0
	2010	238	150	11 [50]	2 **(0.8)**	0	0	n.d.	n.d.	n.d.
*R2	2009	213	145	14 [63]	2 **(0.9)**	0	1 [2] **(1.6-3.2)**	9 **(4.2)**	5 **(3.4)**	1 [1] **(1.6)**
**I1**	2009	328	156	19 [91]	0	0	0	16 **(4.9)**	11 **(7.1)**	2 [10] **(2.2-11.0)**
	2010	203	142	14 [70]	1 **(0.5)**	0	0	n.d.	n.d.	n.d.
*I2	2009	344	143	24 [109]	2 **(0.6)**	0	0	34 **(9.9)**	7 **(4.9)**	5 [25] **(4.6-22.9)**
*A	2009	166	83	19 [91]	0	0	0	10 **(6.0)**	8 **(9.6)**	4 [20] **(4.4-22.0)**
**B**	2009	235	141	21 [90]	3 **(1.3)**	2 **(1.4)**	0	21 **(8.9)**	14 **(9.9)**	0
	2010	162	150	5 [15]	0	2 **(1.3)**	0	n.d.	n.d.	n.d.

**month**										

*April	2009	330	85	3 [13]	0	0	0	15 **(4.5)**	2 **(2.4)**	0
**May**	2009	540	211	12 [51]	4 **(0.7)**	0	0	34 **(6.3)**	19 **(9.0)**	1 [5] **(2.0-9.8)**
	2010	300	150	6 [30]	0	1 **(0.7)**	0	n.d.	n.d.	n.d.
**June**	2009	531	261	42 [202]	3 **(0.6)**	3 **(1.1)**	0	50 **(9.4)**	25 **(9.6)**	2 [10] **(1.0-5.0)**
	2010	300	150	8 [39]	4 **(1.3)**	0	1 [5] **(2.6-12.8)**	n.d.	n.d.	n.d.
**July**	2009	463	195	26 [115]	3 **(0.6)**	0	0	49 **(10.6)**	15 **(7.7)**	3 [15] **(2.6-13.0)**
	2010	257	150	9 [37]	1 **(0.4)**	1 **(0.7)**	0	n.d.	n.d.	n.d.
**August**	2009	396	228	43 [201]	3 **(0.8)**	0	1 [2] **(0.5-1.0)**	29 **(7.3)**	14 **(6.1)**	5 [21] **(2.5-10.4)**
	2010	187	142	14 [66]	3 **(1.6)**	0	0	n.d.	n.d.	n.d.
**September**	2009	235	210	40 [192]	0	0	0	11 **(4.7)**	6 **(2.9)**	5 [25] **(2.6-13.0)**
	2010	98	150	19 [78]	0	0	0	n.d.	n.d.	n.d.

**total**	2009	2495	1190	166 [774]	13 **(0.5)**	3 **(0.3)**	1 [2] **(0.1-0.3)**	188 **(7.5)**	81 **(6.8)**	16 [76] **(2.1-9.8)**

	2010	1142	742	56 [250]	8 **(0.7)**	2 **(0.3)**	1 [5] **(0.4-2.0)**	n.d.	n.d.	n.d.

**species**					19 × *B*. EU1 1 × *B. div/cap* 1 × *B. gibs*-l.	4 × *B*. EU1 1 × *B. div*	2 × *B*. EU1	180 × *R. helv* 7 × *R. mon *IrR 1 × *R. mon*	77 × *R. helv* 4 × *R. mon *IrR	15 × *R. helv* 1 × *R. mon *IrR

*no investigation in 2010; A: adult ticks, N: nymphs, L: larvae; n.d.: not detected; s.f.: sequence failed

Species: *B*. EU1: *Babesia *sp. EU1; *B. div*: *Babesia divergens*; *B. div/cap*: *Babesia divergens/capreoli*; *B. gibs*-l.: *B. gibsoni*-like

*R. helv*: *Rickettsia helvetica*; *R. mon *IrR: *Rickettsia monacensis *IrR/Munich; *R. mon*: *Rickettsia monacensis*

GenBank analysis of the *Babesia *spp. positive samples (n = 28) revealed 25 samples with a 447 bp sized sequence, 100% homologous to sequences of *Babesia *sp. EU1 from *I. ricinus*, roe deer and human patients from several European countries [GenBank: GQ856650, GU647159, FJ215873, EF185818 and AY046575]. One more positive sample showed 100% identity to a sequence of *B. capreoli *from roe deer in France [GenBank: FJ944828] and also to a sequence of *B*. *divergens *from reindeer in the UK [GenBank: AY098643]. The sequence was not discriminating enough to allow a clear species identification. One sequence was 100% homologous to several sequences of *B. divergens *in cattle, human patient and *I. ricinus *[GenBank: FJ944826, FJ944823, AY789076, AY648876, AY648871]. However, only 303 bp of this sequence could be evaluated. The last sequence (445 bp) was 100% identical to *B. gibsoni *isolates from Asia and the USA [GenBank: AF271082, AF271081] as well as from a dog in Spain [GenBank: AY278443]. Sequences from *Babesia *sp. EU1, *B. divergens *and *B. gibsoni*-like were deposited in GenBank with the following accession nos.: *Babesia *sp. EU1: [GenBank: JN036420-JN036426], *B. divergens*: [GenBank: JN036427], *B. gibsoni*-like: [GenBank: JN036428].

### Molecular detection of *Rickettsia *spp

4,459 ticks, collected in 2009, were screened for the occurrence of rickettsial DNA. An overall prevalence of 6.4 to 7.7% (n = 285, including 16 pools of 76 larvae) was observed. Adult ticks were infected with 7.5%, nymphs with 6.8% and larvae in a range of 2.1 to 9.8% (Table 2). The differences between the developmental stages were not significant. The minimum prevalences in April (4.0%, p < 0.05) and September (3.5-6.6%, p < 0.05) were significantly lower in comparison to the prevalences observed in June (7.7 to 8.6%) and July (8.7 to 10.2%). One sampling site (M1) showed a significantly higher minimum prevalence than sampling site R2 (p < 0.01) as well as sampling sites M2, R1 and I1 (p < 0.05). However, the effects for these sampling sites are expected to disappear, if more than one larva per positive pool was assumed to be infected.

285 samples yielded bands of the appropriate size in the PCR, targeting the *gtlA*- gene of *Rickettsia *spp.. Sequence analysis showed in 268 samples 100% homology to sequences of *R. helvetica *[GenBank: HM371185, EU359287- EU359297, EU596563, EF392725, DQ821857, AM418450, DQ910785]. All 268 samples were 100% identical to each other. The remaining 17 samples, identified as *R. monacensis *and unspecified *Rickettsia *spp. were further analysed with a PCR, targeting the *ompB *gene. 12 samples were then specified as *R. monacensis *strain IrR/Munich, 4 additional samples as *R. helvetica *(100% homolog to [GenBank: HQ232251, GU324465, AF123725]) and 1 sample yielded no band in the *ompB*-PCR. It was 100% homologous to sequences of unspecified *R. monacensis *in *gltA*-gene sequence analysis [GenBank: EU853831, FJ009429]. The *R. monacensis *strain IrR/Munich sequences were 99-100% identical to each other, with a difference in one nucleotide position (100% homolog to [GenBank: EU330639] and [GenBank: EU330640], respectively).

Exemplary sequences of *R. helvetica *were chosen for the 268 sequences of 100% identity with the following accession numbers: *gltA*-gene: [GenBank: JN036402-JN036410]; *ompB*-gene: [GenBank: JN036411-JN036412]; Sequences of *R. monacensis *strain IrR/Munich were deposited in GenBank [GenBank: JN036413-JN036417 and GenBank: JN036418-JN036419].

### Molecular detection of *Bartonella *spp

4,459 ticks from 2009 were screened for the occurrence of DNA of *Bartonella *spp.. Five samples (0.1%) showed an appropriate band. However, sequence analysis of the PCR product revealed only 85 to 100% similarity to various sequences of uncultivated *Bartonella *spp. Further investigations to amplify a sequence of the *16S rRNA*-, *gltA- *and *16S-23S*-gene of *Bartonella *spp. and consequently species identification failed (data not shown).

### Coinfections

4,459 samples (3,685 for *A. phagocytophilum*) were screened for coinfections of *Babesia *spp., *Rickettsia *spp., *A. phagocytophilum *and *Bartonella *spp. in 2009 and 1,884 samples for *Babesia *spp. and *A. phagocytophilum *in 2010. The detailed results for *A. phagocytophilum *will be reported elsewhere [Schorn et al. unpublished]. In 2009, 0.7% of the samples (n = 27) were positive for *R*. *helvetica *and *A. phagocytophilum*, 0.1% (n = 4, including 3 × *Babesia *sp. EU1 and 1 × *B*. *gibsoni*-like) for *Babesia *spp. and *A. phagocytophilum *and 0.04% (n = 2, including *Babesia *sp. EU1 and *B. divergens*) for *Babesia *spp. and *R*. *helvetica*. No triple or quadruple infections of ticks were detected in 2009. In 2010, no coinfections between *Babesia *spp. and *A. phagocytophilum *were observed.

## Discussion

The results of this study show that tick-borne pathogens can occur not only in natural woodlands, but also in recreational urban areas. This demonstrates the importance of systematic investigations of different habitat types to broaden the understanding and knowledge about these pathogens and their tick vector.

The evidence of *Babesia *sp. EU1 in *I. ricinus *collected in Bavarian public parks adds a new species to the current knowledge on tick-borne *Babesia *spp. in Germany. Previous studies demonstrated the occurrence of *B. microti *and *B. divergens *in ticks with a predominance of *B. divergens *in Southern Germany and almost equal distribution of *B. divergens *and *B. microti *in Central Germany [13,58,59]. On the contrary, in this study *Babesia *sp. EU1 was the dominating *Babesia *species. However, not only the distribution of *Babesia *species differs geographically; the infection rates of ticks in Southern Germany are also lower (about 1%) than in Central Germany (up to 10.7%) [13,58-60]. The overall prevalence of 0.4 to 0.5% for *Babesia *spp. detected in this study was comparable to these results. A similar variability of both the *Babesia *spp. prevalences and the arrangement of species within a country could also be recognized in Austria (local variations: 0 to 100%) [19], in Switzerland (0.7 to 4%) [12,61] and in Poland (0.6 to 16.3%) [17,20,62-64]. Such a local clustering of *Babesia *spp. may be associated with the tick density in the investigated habitat. The maintenance of the protozoan life cycle by transovarial transmission, which has been demonstrated for *B. divergens *and *Babesia *sp. EU1, is more efficient in areas with a high tick density [19,61,65,66]. Moreover, most of the *Babesia *spp. positive ticks were found on sampling sites M3 and B in this study. This is an interesting fact, because both areas are known to harbor a permanent population of large mammals like roe- and red deer in contrast to the other sites where those animals are missing or only transiently present. Beside *I. ricinus*, cervids, like roe deer, are discussed as potential reservoir for *B. divergens *and *Babesia *sp. EU1 [67-69].

It is noteworthy that no *B. microti *were detected in these public parks. Microtine rodents and shrews are suggested to be reservoir hosts for *B. microti *[70,71]. Our findings are supported by the fact that another study from Southern Germany showed that rodents of the family Muridae were not infected with *B. microti*, whereas rodents of the family Arviculidae were infected at a rate of 1.6% [72]. Therefore a possible explanation for the lack of *B. microti *in *I. ricinus *in public parks may be the occurrence and arrangement of rodent and insectivore species, which can be dissimilar to woodland areas [73].

A very interesting result is the finding of a 445 bp sized *18S rRNA *gene sequence, 100% homologous to existing sequences of *B. gibsoni *in GenBank. Although this is not evidence for the occurrence of *B. gibsoni *in *I. ricinus*, it is a hint that *I. ricinus *could potentially harbor *Babesia *of the gibsoni-complex. *B. gibsoni*, a small Babesia and one of the agents causing canine babesiosis, is distributed all over the world, but only sporadically found in Europe. Recently, Zahler et al. [74] postulated that *B. gibsoni *has to be divided in two different lineages with isolates from Asia belonging to the *Babesia *spp. senso stricto clade and isolates from the USA being more related to *Theileria *spp. So far, the classification of the European isolates *of B. gibsoni *has not been fully determined [75]. The sequence obtained in this study was in the amplified part 100% homologous to sequences of both lineages, thus no specification was possible. Further studies will help to clarify if *I. ricinus *could potentially serve as competent vector for additional *Babesia *spp. than assumed so far [62]. This would bring a new aspect into the epidemiology of canine babesiosis in Central Europe and offer a possible explanation for two clinical cases of canine babesiosis in Southern Germany assumedly leaded back to *B. gibsoni*. These two dogs with no history of travelling, blood transfusion or dogfights showed *Babesia*-like parasites in blood smear examination and 100% identity to *B. gibsoni *(Asian genotype) in sequencing of the *18S rRNA *gene [76].

The overall prevalence of *Rickettsia *spp. detected in Bavarian public parks in this study, which is 6.4 to 7.7%, was comparable to results of previous studies in Germany. In Southern Germany prevalences of 5.3 to 12% were previously detected, whereas prevalences up to 14.7% were recorded in eastern federal states of Germany [13,58,77-81]. The occurrence and arrangement of *Rickettsia *species (95% *R. helvetica *and 5% *R. monacensis*) were also similar to previous results from Germany [77,79,82]. The distribution of *R. monacensis *in *I. ricinus *seems to show geographical distinctions between Northern and Southern Europe. In Southern and Eastern European countries *I. ricinus *was much more infected with *R. monacensis *than with other spotted fever group Rickettsiae [83,84], in Central Europe *R. monacensis *was only sporadically found beside the main agent *R. helvetica *[16,79] and in Northern Europe *R. helvetica *exclusively was found in *I. ricinus *[85,86].

Rickettsial prevalences vary geographically in European countries from 1.5 to > 40.6% [32,86]. Beside this, local, seasonal and annual prevalence variations of *Rickettsia *spp. were seen as well as differences between habitat-types [32,87,88]. In this study local prevalence differences (between 3.6 and 12.2% depending on sampling site) as well as seasonal variations (highest prevalences in June and July) were also demonstrated. To date, the reasons for these variations are not well understood, but a correlation between tick density and the occurrence for *Rickettsia *spp. is suggested, because of the reservoir role of the ticks in the maintenance of rickettsial life cycle [87].

In this study the occurrence of *Bartonella *spp. in *I. ricinus *could not be definitively demonstrated, because sequence similarity of > 85% is not sufficient for specification. Three potential reasons could be the decisive factor for this result: (i) The sensitivity of the method used with ticks as template tissue; the amount of detectable *Bartonella *DNA may have been too low in the individually investigated ticks. (ii) Urban areas were exclusively investigated in this study and may not serve as suitable habitat for the maintenance of the life cycle of *Bartonella *spp. in ticks, because of a lack of competent reservoir host. (iii) The tick *I. ricinus *may not play an important role in the transmission of *Bartonella *spp. [39,89].

Coinfections with different pathogens were recognized in 34 ticks (0.9%) in 2009. Most of them (n = 27) were infections with *R. helvetica *and *A. phagocytophilum*, i.e. 0.7% of all investigated ticks. This is comparable with previous studies; 0.5 to 1% of ticks were infected with *Rickettsia *spp. and *A. phagocytophilum *in different European countries [77,84,86,90]. On the contrary, lower loads of double infections were also observed in other studies with 0.1% in Luxembourg and with 0.2% in Germany [16,58]. However, this can be traced back to the fact that *A. phagocytophilum *was only detected with low prevalences (1.9% and 1% accordingly), whereas in the ticks investigated in this study *A. phagocytophilum *was detected with an average prevalence of 9.5% in 2009 and 6.6% in 2010 [Schorn et al. unpublished data]. Furthermore, coinfections with the other pathogens were sporadically found in this study (0.04 to 0.1%). Double infections with *Babesia *spp. and *A. phagocytophilum *were previously found in 0.3% of ticks in Germany and 0.7 to 2% in Poland [13,50,91]. Coinfections between *Babesia *spp. and *Rickettsia *spp. were previously detected in Italy, Luxembourg and Germany with 0.1 to 0.6% [13,16,92]. Additional data about the occurrence of *Borrelia burgdorferi *sensu lato in public parks would enrich the current knowledge on the dynamics of coinfections in *I. ricinus *in urban areas. Previous epidemiological studies from Germany on *Borrelia burgdorferi *sensu lato show that 1.0% of all investigated ticks in the 'English Garden' in Munich (sampling site M2 in this study) were coinfected with *A. phagocytophilum *as well as 0.2% with *Babesia *spp., whereas the most prevalent coinfection in woodland areas was with *Babesia *spp. (2.6%) and none was detected with *A. phagocytophilum *[13,60].

## Conclusions

This study shows that several pathogens including *Babesia *spp., *Rickettsia *spp. and *A. phagocytophilum *are present in ticks in public parks in Germany and coinfections of these agents comparable to other habitats and other European countries were demonstrated. Even though the level of pathogenicity of these detected pathogens is not yet fully known, it can be assumed that people visiting public parks and their companion animals may be at risk when coming into contact with these agents. Therefore, visitors to urban public parks, public health officials and physicians should be made aware of the potential risk of tick bites and the possible medical consequences on the urban population.

## Competing interests

The authors declare that they have no competing interests.

## Authors' contributions

SS performed the experiments, analyzed the data and drafted the manuscript. CS conceived and designed the experiments and gave critical proposals in every step of development. HR and MM performed the statistical analysis. KP conceived the idea of the study. CS and KP critically reviewed the manuscript. All authors read and approved the final manuscript.
